# Supplementary material for: Key link between iron and the size structure of three major mesoplanktonic groups in the upper ocean
Source: Nat Commun. 2026 Jul 14;17:8645. doi: 10.1038/s41467-026-75355-4 (PMC13486666; doi:10.1038/s41467-026-75355-4)
Supplement: Supplementary file 1 [file 41467_2026_75355_MOESM1_ESM.pdf]

Supplementary information:

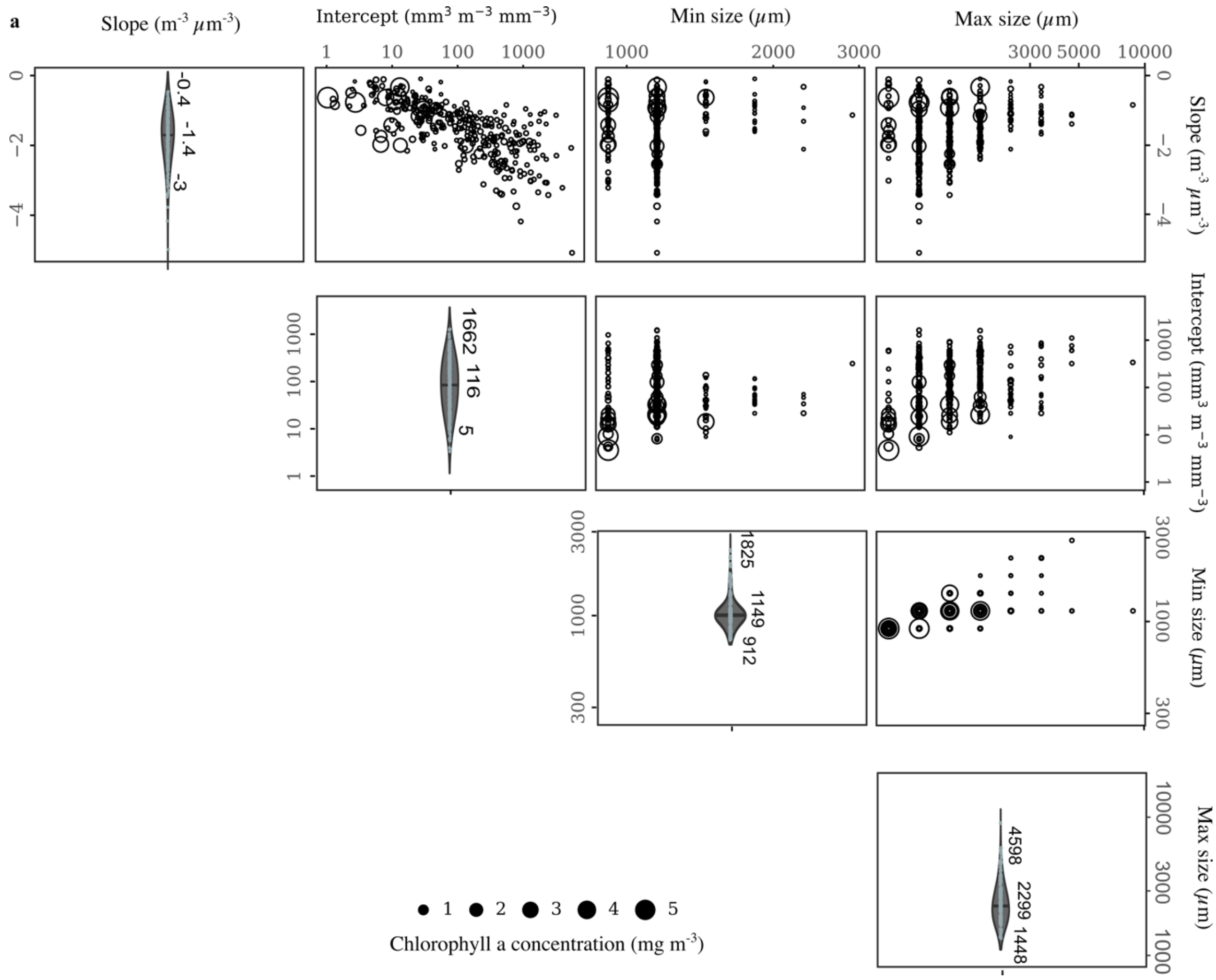

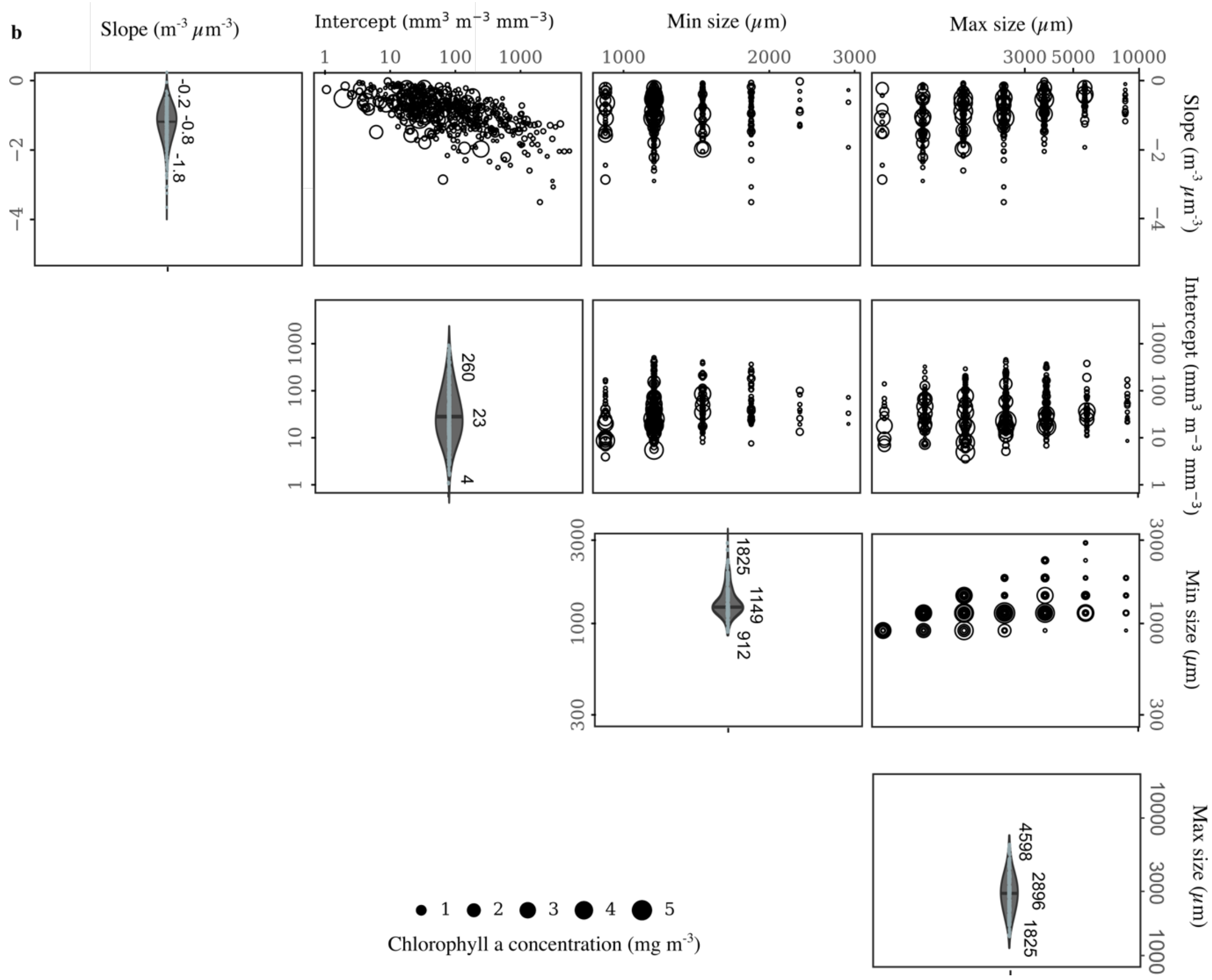

Caption bellow

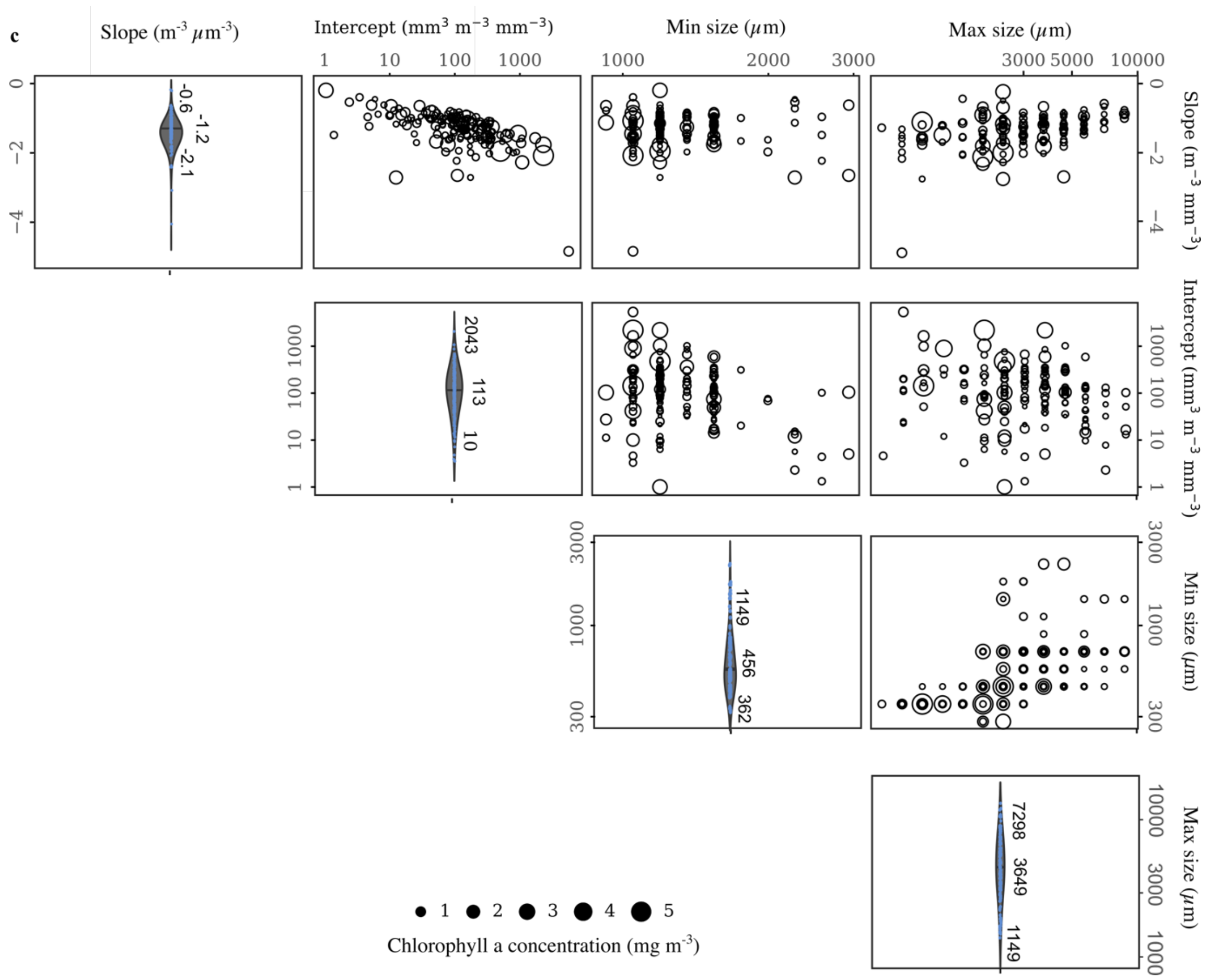

Supplementary Figure 1. Distribution (diagonal) and correlation within spectral parameters (intercept, slope, and size range) of three mesoplankton groups. a Colonial N_2-fixers (n=603). **b** Rhizarians (n=572). **c** Crustaceans (n=188). Datapoints vary in size according to the average chlorophyll-a concentration observed in satellite products in the same spatio-temporal bin. The distribution of spectral parameters is shown with violin plots on the diagonal.

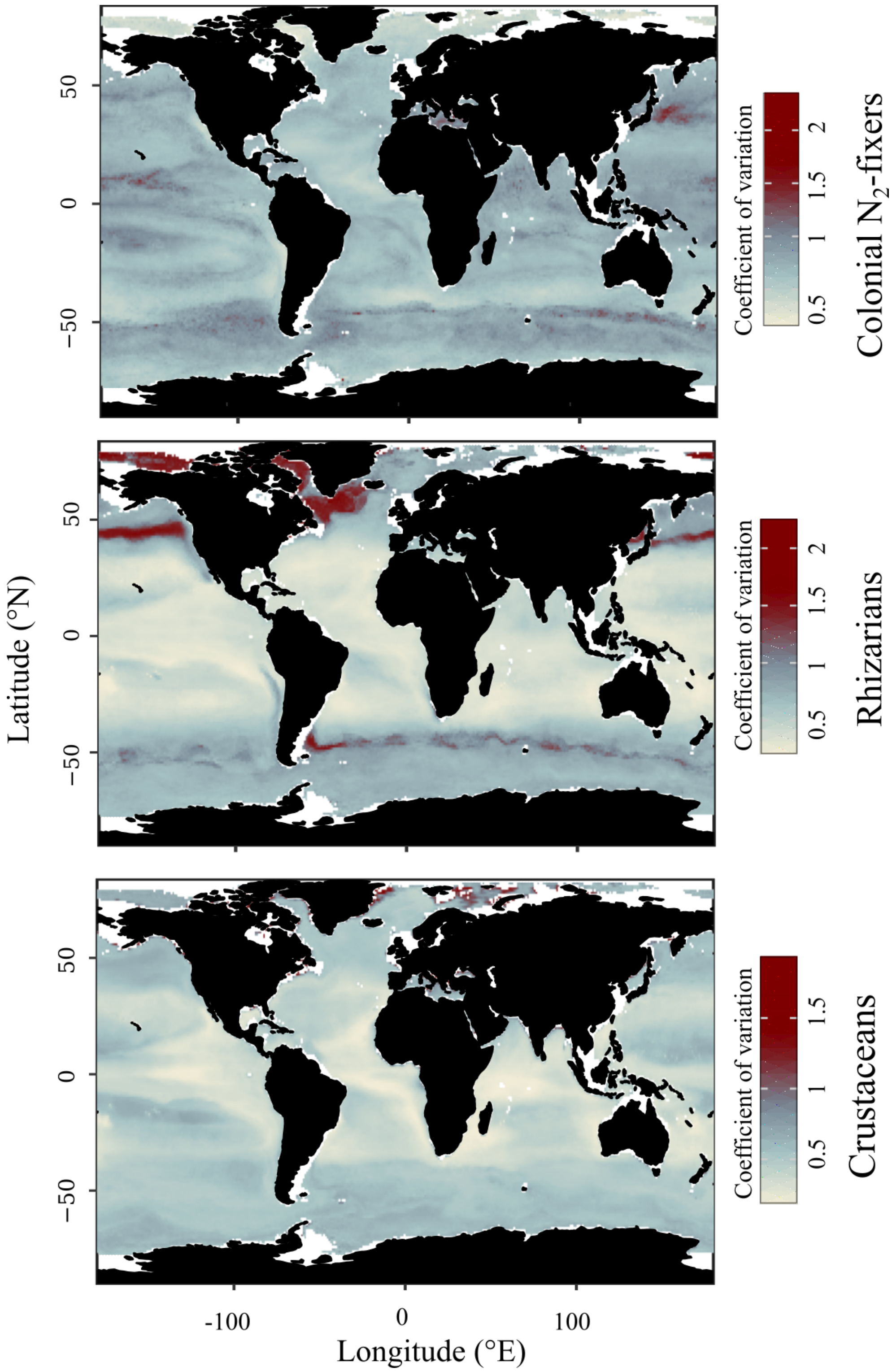

Supplementary Figure 2. Coefficients of variation (standard deviation / mean) for predicted total abundance of the main mesoplankton groups obtained from 50 models fitted to bootstrapped (random sampling with replacements) observations. These represent the uncertainty of model predictions for colonial N_2-fixers (top panel), Rhizarians (middle panel) and Crustaceans (bottom panel) spectral biogeography.

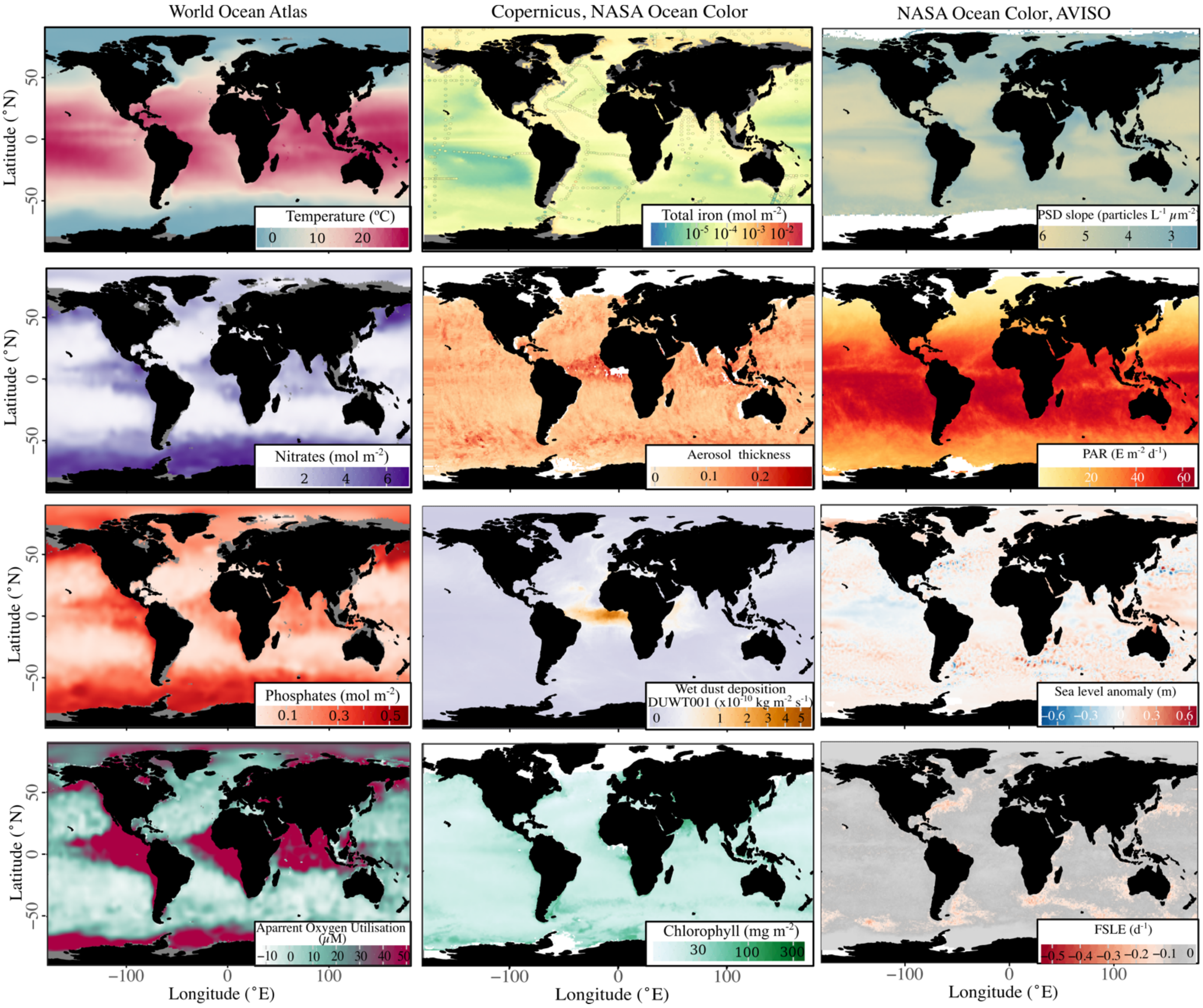

Supplementary Figure 3. Environmental factors used to predict the spectral biogeography of the main mesoplankton groups. Set of average (for January) environmental factors included as explanatory variables to predict the size distribution of colonial N_2-fixers, Rhizarians and Crustaceans globally. Variables include average temperature and integrated concentrations of nitrates, phosphates, silicates (not shown given its strong correlation with nitrates), and apparent oxygen utilization, along with mixed layer depth (MLD) from World Ocean Atlas. The concentration of diverse iron pools were approximated by satellite (wet dust deposition and aerosol optical thickness, representative of the surface layer) or biogeochemical model (0-200 m total iron) products. Total iron concentrations measured *in situ* as part of the GEOTRACES program (colored dots, downloaded at https://geotraces.webodv.awi.de/service/) are superimposed on top of the model PISCES continuous predictions to validate the product used in our study. Phytoplankton concentration, productivity, and composition was approximated by integrated chlorophyll-a concentration (CHL), net primary production (not shown), particle size distribution slope (PSD slope), and fractions of pico-, nano- and microphytoplankton (not shown since their relative proportion affect directly PSD slopes) distributed by NASA, alongside daily photosynthetically available radiance (PAR). Mesoscale activities were indicated by two AVISO products: Sea level anomaly (SLA), indicative of cyclonic or anticyclonic eddies under negative or positive anomaly, and finite size Lyapunov exponent (FSLE), marking the divergence rate of water parcels in close proximity.

Supplementary Table 1. Spearman correlation coefficients between environmental factors and the integral of mesoplankton size spectra calculated at the finest taxonomic resolution. Environmental factors are ranked according to their feature importance in model fits (ranking in parenthesis). P-values are indicated in italics below the correlation coefficient

Rank:	Environmental factors:	Spearman correlation coefficients: P-values	
	Colonial N_2-fixers:	Puff	Tuff
1	Temperature (°C)	0.37*(3)<br>*0.02*	0.35***(2)<br>*0*
2	Phosphates (mol m^{-2})	-0.07(7)	-0.08(10)
3	Aerosol (unitless)	0.46***(6)<br>*0*	0.28**(5)<br>*0*
4	Total iron (mol m^{-2})	0.28***(4)<br>*0*	0.28***(3)<br>*0*
5	Wet dust deposition (kg m^{-2} s^{-1})	0.33***(2)<br>*0*	0.37***(1)<br>*0*
6	Photosynthetic Available Radiation (E m^{-2} d^{-1})	-0.25**(10)<br>*0.02*	-0.24*(7)<br>*0.02*
7	Mixed Layer Depth (m)	0.21*(8)<br>*0.01*	0.04(11)
8	Apparent Oxygen Utilisation (mol m^{-2})	0.11**(16)<br>*0.00104*	0.11**(12)<br>*0.002*
9	Silicates (mol m^{-2})	-0.11(5)	-0.12**(4)<br>*0.001*
10	PSD slope (L^{-1} μm^{-2})	0.12(18)	0.16(18)
11	Nitrates (mol m^{-2})	-0.14*(9)<br>*0.05*	-0.09*(8)<br>*0.03*
12	Picophytoplankton (fraction)	0.12(17)	0.16(17)
13	Microphytoplankton (fraction)	-0.15(15)	-0.19(15)
14	Sea Level Anomaly (m)	-0.01(12)	0.05(13)
15	CHL (mg m^{-2})	-0.07(14)	-0.08*(14)<br>*0.03*
16	Finite Size Lyapunov Exponent (d^{-1})	0.11(13)	0.13(6)
17	Nanophytoplankton (fraction)	-0.15(16)	-0.21***(16)<br>*0.00011*
18	Net Primary Production (mg m^{-2} d^{-1})	-0.15(11)	-0.20*** (9)<br>0.00001
Extra	GEOTRACES† dissolved iron (mol m^{-2})	-0.09(-0.12)	0.09***(-0.13)<br>*0.0007*

† Based on GEOTRACES (v. 2021 downloaded at https://geotraces.webodv.awi.de/service/IDP2021_v2%3EGEOTRACES_IDP2021_Seawater_Discrete_Sample_Data_v2) surface dissolved iron concentration measured from Niskin bottles averaged by Longhurst regions and month.

Significance levels for Spearman correlation coefficients *: 0.05 **: 0.01 ***: 0.001

	Rhizarians:	Acantharia	Collodaria	Phaeodaria	Foraminifera
1	Temperature (°C)	0.17***(1) *0.0004*	0.21***(7) *0.0001*	-0.13*(14) *0.01*	-0.01(7)
2	Wet dust deposition (kg m^{-2} s^{-1})	0.02(6)	0.28***(3) *0.0003*	-0.05(7)	-0.13**(14) *0.002*
3	Apparent Oxygen Utilisation (mol m^{-2})	0.14*(4) *0.05*	0.04(5)	-0.16***(4) *0*	-0.03(13)
4	Photosynthetic Available Radiation (E m^{-2} d^{-1})	0.15***(13) *0.0008*	0.2***(12) *0.0005*	0.15**(11) *0.003*	0.05(9)
5	Total iron (mol m^{-2})	0.01(12)	-0.03(11)	0.1(3)	-0.22***(12) *0*
6	Finite Size Lyapunov Exponent (d^{-1})	0.004(10)	-0.05(10)	-0.05(1)	-0.02(2)
7	Mixed Layer Depth (m)	-0.2***(7) *0*	0.06(8)	-0.26***(9) *0*	0.004(15)
8	Sea Level Anomaly (m)	0.09*(11) *0.03*	0.11*(9) *0.01*	0.22***(12) *0*	0.06(17)
9	Phosphates (mol m^{-2})	0.08(3)	-0.01(6)	0.30***(6) *0*	0.18***(3) *0.00016*
10	PSD slope (L^{-1} μm^{-2})	-0.01(17)	-0.01(17)	-0.19***(15) *0*	0.04(11)
11	Nitrates (mol m^{-2})	0.22**(2) *0.001*	-0.10*(4) *0.01*	0.29***(18) *0*	-0.01(5)
12	Integrated CHL (mg m^{-2})	0.02(15)	-0.03(15)	0.23***(13) *0*	-0.01(10)
13	Silicates (mol m^{-2})	0.03(14)	-0.24***(1) *0.00002*	0.29***(2) *0*	0.04(6)
14	Picophytoplankton (fraction)	-0.007(18)	-0.01(18)	-0.19***(16) *0*	0.04(16)
15	Nanophytoplankton (fraction)	0.04(16)	0.01(16)	0.18***(17) *0*	-0.03(18)
16	Aerosol (unitless)	0.43***(8) *0*	0.51***(2) *0*	0.11*(10) *0.01*	0.21**(4) *0.006*
17	Microphytoplankton (fraction)	0.007(5)	0.01(14)	0.22***(8) *0*	-0.04(1)
18	Net Primary Production (mg m^{-2} d^{-1})	-0.13 (9)	-0.03 (13)	0.18*** (5) *0*	0.18*** (8) *0.00001*
Extra	GEOTRACES[†] dissolved iron (mol m^{-2})	0.05 (-0.05)	0 (0.15)	0.64***(0.22) *0.0008*	0.53***(0.19)

		Crustaceans: Malacostraca	Ostracoda	Copepoda	Carnivore	Omnivore
1	Total iron (mol m^{-2})	0.15***(6)<br>*0.00004.*	0.32***(4)<br>*0.00019*	0.26***(1)<br>*0*	-0.13(8)	0.24***(5)<br>*0.0003*
2	Photosynthetic Available Radiation (E m^{-2} d^{-1})	-0.14*(5)<br>*0.027*	0.01(9)	0.19**(10)<br>*0.002*	0.04(12)	-0.15(15)
3	Sea Level Anomaly (m)	-0.08(7)	-0.16*(6)<br>*0.04*	-0.2**(5)<br>*0.0013*	-0.02(5)	-0.33**(11)<br>*0.00035*
4	Temperature (°C)	-0.15***(3)<br>*0.000121*	0.34***(1)<br>*0.00016*	-0.2***(3)<br>*0*	0.12*(9)<br>*0.01*	-0.39***(3)<br>*0.00010*
5	Mixed Layer Depth (m)	0.19**(11)<br>*0.003*	-0.02(11)	-0.4***(9)<br>*0*	0.04(6)	0.4(8)
6	PSD slope (L^{-1} μm^{-2})	-0.13(15)	0.04(17)	-0.29***(14)<br>*0.00003*	0.11(13)	-0.18***(2)<br>*0.00065*
7	Integrated CHL (mg m^{-2})	0.21**(16)<br>*0.005*	0.19*(2)<br>*0.01*	0.43***(4)<br>*0*	0.02(10)	0.36***(7)<br>*0.000136*
8	Apparent Oxygen Utilisation (mol m^{-2})	0.03(17)	-0.00(16)	0.19***(11)<br>*0.00016*	-0.03(14)	0.14(14)
9	Aerosol (unitless)	0.17*(12)<br>*0.04*	0.2*(3)<br>*0.05*	-0.05(12)	0.14*(11)<br>*0.045*	-0.27**(10)<br>*0.0048*
10	Phosphates (mol m^{-2})	0.03(4)	-0.16*(7)<br>*0.05*	-0.09(6)	0.06(4)	0.2***(13)<br>*0.0007*
11	Finite Size Lyapunov Exponent (d^{-1})	-0.24**(14)<br>*0.004*	-0.16*(5)<br>*0.035*	-0.04(13)	-0.03(3)	0.17(6)
12	Wet dust deposition (kg m^{-2} s^{-1})	-0.04(8)	0.04(15)	0.2**(8)<br>*0.002*	-0.15*(2)<br>*0.01*	-0.61***(9)<br>*0.0003*
13	Nitrates (mol m^{-2})	0.16***(1)<br>*0.00053*	-0.02(12)	0.04(15)	-0.00(15)	0.23***(18)<br>*0*
14	Picophytoplankton (fraction)	-0.14*(18)<br>*0.03*	0.04(18)	-0.29***(7)<br>*0.00005*	0.11(18)	-0.43***(4)<br>*0.00015*
15	Nanophytoplankton (fraction)	0.14*(13)<br>*0.02*	-0.04(13)	0.28***(2)<br>*0.00009*	-0.12(17)	0.43***(12)<br>*0.00014*
16	Silicates (mol m^{-2})	0.15**(2)<br>*0.005*	-0.03(8)	0.10(17)	- 0.02(1)	0.27***(16)<br>*0.00027*
17	Microphytoplankton (fraction)	0.15*(10)<br>*0.02*	-0.11(14)	0.28***(18)<br>*0.00008*	-0.12(16)	0.44***(17)<br>*0.00012*
18	Net Primary Production (mg m^{-2} d^{-1})	0.15* (9)<br>*0.04*	-0.21** (10)<br>*0.002*	0.25** (16)<br>*0.0018*	0.0(7)	0.25 (1)
Extra	GEOTRACES† dissolved iron (mol m^{-2})	0.09(0.11)	0.15(0.20)	0.20***(-0.40)<br>*0.0009*	0.01 (-0.04)	0.25*(-0.26)<br>*0.018*

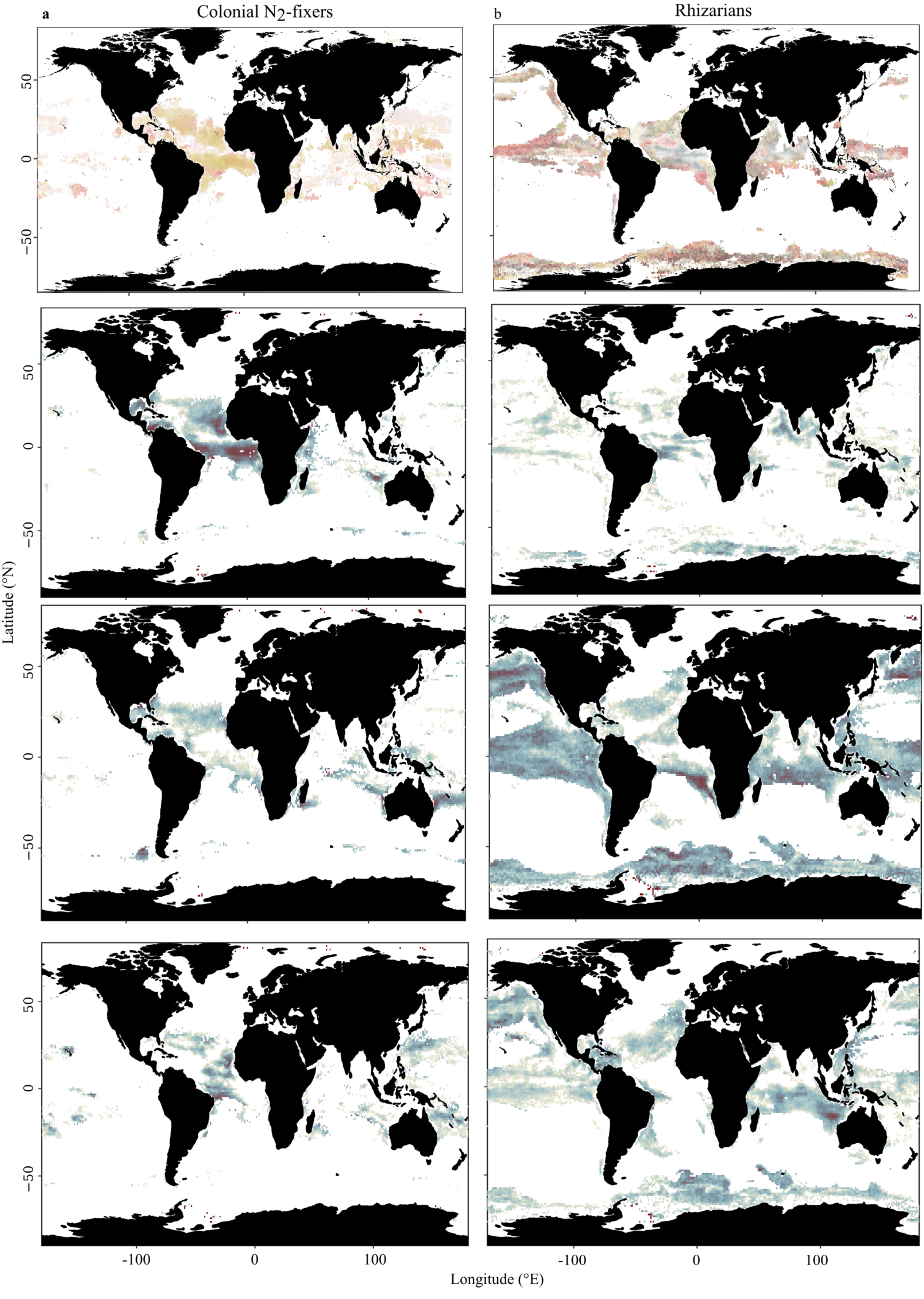

Caption bellow

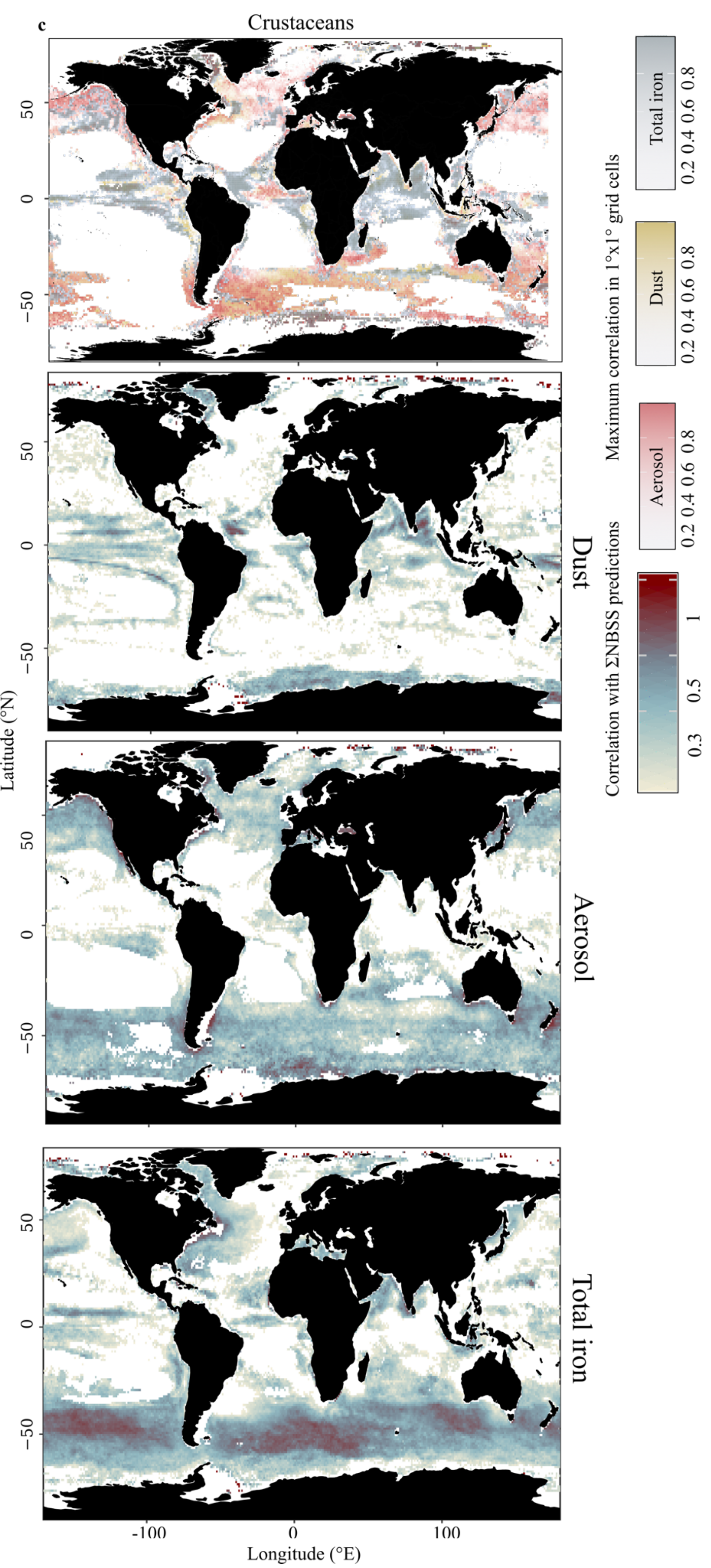

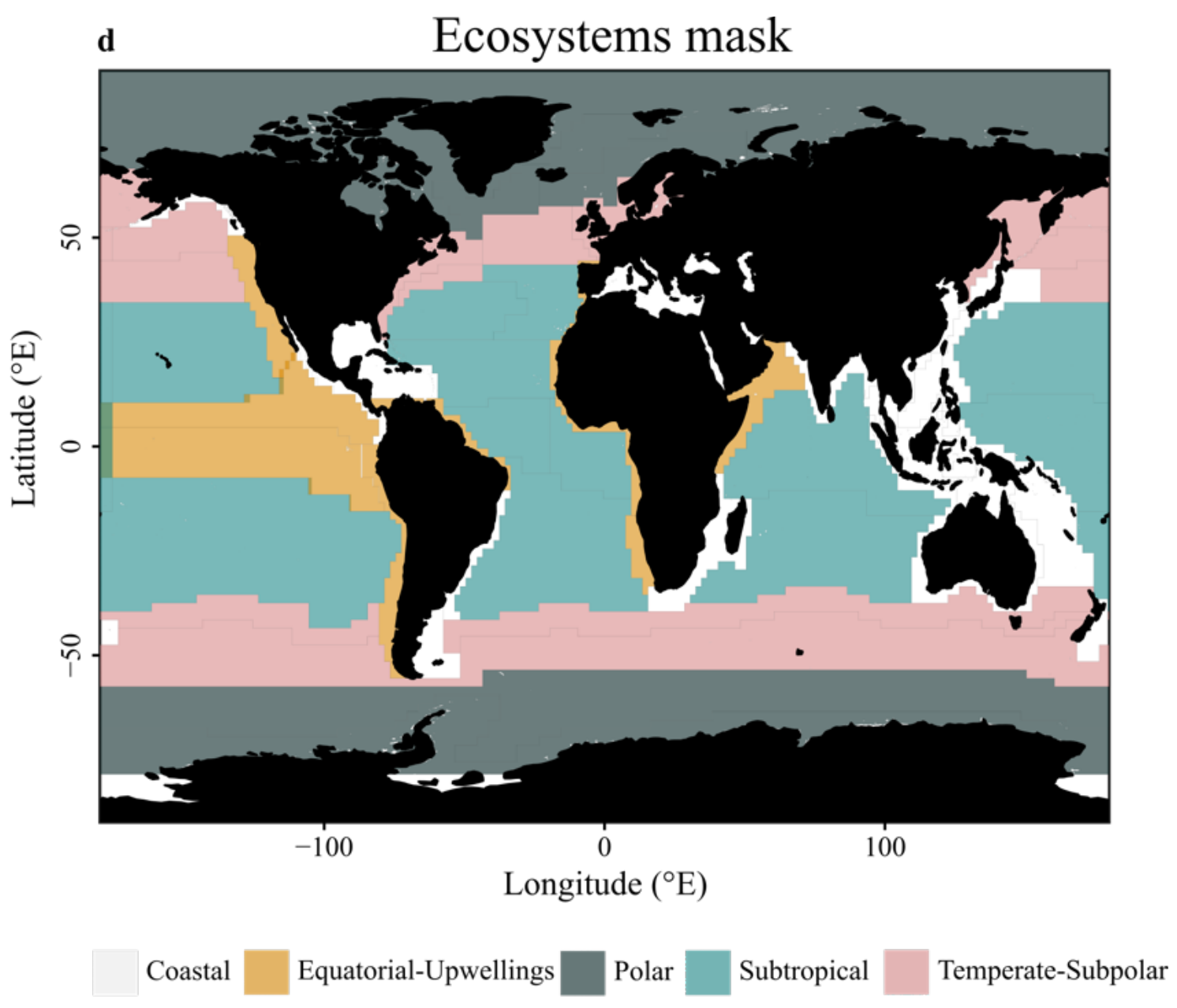

Supplementary Figure 4. Correlations between iron proxies and predictions of NBSS integral in 1°x1° grid cells. a Identification of main iron proxy (top panel) based on the maximum correlation coefficients between iron proxies (dust, aerosol, or total iron) and predictions of colonial N_2-fixers NBSS integral in 1°x1° grid cells. **b** Identification of main iron proxy (top panel) based on the maximum correlation coefficients between iron proxies (dust, aerosol, or total iron) and predictions of Rhizarians NBSS integral in 1°x1° grid cells. **c** Identification of main iron proxy (top panel) based on the maximum correlation coefficients between iron proxies (dust, aerosol, or total iron) and predictions of Crustaceans NBSS integral in 1°x1° grid cells. **d** Mask of ecosystems (Equatorial-Upwellings: orange, Polar: green, Subtropical: blue, Temperate-Subpolar: pink) used to plot seasonal trends of NBSS predictions in association with the main iron proxy. The main proxy is identified in each ecosystem / ocean basins by calculating the number of bins associated to each iron proxy. The proxy with the maximum number of bins in a given ecosystem/ocean basin is considered the main iron pool.
