# Supplementary material for: Key link between iron and the size structure of three major mesoplanktonic groups in the upper ocean
Source: Nat Commun. 2026 Jul 14;17:8645. doi: 10.1038/s41467-026-75355-4 (PMC13486666; doi:10.1038/s41467-026-75355-4)
Supplement: Supplementary file 3 [file 41467_2026_75355_MOESM3_ESM.pdf]

nature portfolio

Peer Review File

Key link between iron and the size structure of three main mesoplanktonic groups in the upper ocean

Corresponding Author: Dr Mathilde Dugenne

This file contains all reviewer reports in order by version, followed by all author rebuttals in order by version.

Version 0:

Reviewer comments:

Reviewer #1

(Remarks to the Author)
General comments
The manuscript by Dugenne et al. presents an interesting analysis of image-based databases of mesoplankton abundance, biomass and distribution against environmental parameters using various statistical approaches to provide machine learning predictive biogeographical maps. The article is generally well written, and the data and analysis support the conclusions. There are no major issues with the article; three more minor comments relate to the presentation of the data (the figures and sections of the text), an estimation of model error where observations are sparse/non-existent, and consideration of a common depth component to their analysis. Specifically, the article figures include very small text and are difficult to interpret (e.g., what do panels g and h on Fig. 1 represent; are the cake sections and concentric circles providing the same information?).

The second comment relates to an estimate of model confidence where observations (of mesoplankton biomass and size-structure) are sparse or even non-existent. It would be good in the main text to see some estimate of the confidence of the model predictions, i.e., Figure 1a and 1 b highlight that there is very little or no data in the Southern Ocean and Pacific Ocean, whereas Figure 2c presents global data. Using the machine learning tools the authors apply, is there a way to add confidence limits to the areas where the model is predicting outside of its parameter space?

The third comment relates to a consideration of the variable depth components included: the biomass and spectral data are from the upper 200 m; many of the iron proxies and physical mixing metrics describe water-column impacts; whereas the productivity proxies (chlorophyll, nutrients, light, phytoplankton community size composition) are for surface waters only. Have the authors considered whether this introduces bias or removes potential linkages between the mesoplankton biomass and the environmental conditions? For example, does surface nitrate concentration reflect the availability of nitrogen for the community across 200 m? Does incidental irradiance on the sea surface reflect the light availability to the community at depth? Could variability in the depth of the productive layer (i.e. euphotic zone) compensate for low surface water metrics? This is not a major negative comment, but a consideration; that strong relationships are found nevertheless highlights that these are only potential considerations.

Specific comments
Ln 16, is fish production an ecosystem service as with C-export and nutrient supply? What do the authors mean by fish production – a socio-economic concept or grazing / trophic transfer potential? (See also Ln 37)

Ln 17, following a ‘however’ with a ‘how’ is confusing.

Ln 18, not clear at this point of the article as to what ‘spectral biogeography’ is.

Ln 23, how do the authors know that their explanatory variables are comprehensive? Would it not be better to include some indication of what these variables are as to how they are comprehensive?

Ln 43, ‘certain taxa’ and /or certain size classes?

Ln 48, contradictory rather than contradicting.

Ln 49, autotrophic N2-fixers could also include diazotrophic diatoms; better to emphasize that it is only Trichodesmium.

Ln 55-56, individual Trichodesmium cells are only ~10 um so is this paradoxical?

Ln 80, previous line (Ln 66) describes the three groups as keystone, why use main here?

Fig. 3, panel b is confusing with multiple y-axis on the left and right and different colours; please consider a clearer way to present these results.

Ln 209-212, this sentence is long and confusing, introducing copepods and plasticity in their dietary habits and then mentioning the complications of Trichodesmium taxonomy. Consider splitting as difficult to follow the point being made.

Ln 228, How many Phaeodaria and Foraminifera are colonial?

Figs. 4, 5 and 6; the Venn diagrams (a) in each have many (many) numbers and not clear which are the key values to consider. The text in all panels is tiny (especially the correlation coefficients) and the colours in the too pale to clearly see.

Ln 263, does a discussion need a title?

Ln 277, what is considered the best predictor of ecosystem services? The preceding text (Ln 275 onwards) mentions CMIP models, data compilations and plankton size structure, rather than bulk biomass and productivity estimates – which one is the rest of the (long) sentence referring to?

Ln 282, contrasting correlations rather than contrasted correlations.

Ln 302 (and Ln 306), why is upper ocean capitalised (Upper Ocean)?

(Remarks on code availability)

Reviewer #2

(Remarks to the Author)
I find this to be an extremely interesting and potentially important paper that should, ultimately, be published in Nature Communications. However, I do feel uneasy with the manuscript in its present form in that the vertical resolution of the environmental variables included in the models varies greatly.

The mesozooplankton size spectra and distributions studied cover the upper 200 m of the water column, while a number of the environmental variables included in the models are extracted from satellite products which, typically, are derived from surface optical characteristics and, thus, relate to only a very small proportion of the water column (usually around 10m in temperate regions). Only about 25% of global ocean primary production is associated with the surface 10 m and, in tropical regions, up to ~90% of water column productivity occurs below 10 m.

The authors express surprise that the satellite products indicating the relative size distribution of the phytoplankton community did not correlate well with their mesozooplankton spectra/distributions but I honestly would not expect the surface community size distributions to be very important for determining the characteristics of mesozooplankton down to 200 m! Subsurface chlorophyll peaks are dominant features in most of the words oceans and these are often important feeding areas for mesozooplankton. I find the iron correlations intriguing (and a potential game changer in our understanding of plankton ecology. I do not, however, find the lack of convincing correlations with surface phytoplankton community characteristics (and productivity) to support the authors' assertion that iron is more important for determining mesozoopalnkton spectra than the characteristics of the food organisms they depend upon. I believe the authors need to address this discrepancy with respect to vertical scales their environmental variables represent before publication can be recommended.

I am also uneasy with respect to the authors' suggestion that while temperature effects stemming from anthropogenic climate change will likely lead to smaller mesozooplankton, this ecosystem response may potentially be offset by anthropogenic additions of iron to the system. We really cannot from this study derive the relative importance of T and Fe in determining size spectra and, in my experience, conclusions such as those suggested here can be (and often are!) misused by the non-scientific community. I would prefer to see this speculation on the part of the authors removed but that may be a question of personal taste (and too many misrepresentations of my research in the general media).

As far as I can determine (hanging on by my fingernails), the statistics used are robust (except as noted that the environmental variables used do not represent the same scales). There are a few typos and grammatical errors, so careful proof-reading will be necessary if/when the manuscript is accepted for publication. As noted above, however, I do not recommend publication unless/until some consideration is given to the value of using surface proxies to represent pelagic processes to a depth of 200 m.

Katherine Richardson, professor, University of Copenhagen

(Remarks on code availability)

Version 1:

Reviewer comments:

Reviewer #1

(Remarks to the Author)
I commnend the authors on their revised manuscript and have no further comments. The authors have adequately addressed previous concerns about the use of surface metrics to inform on depth-intergrated patterns and provided appropriate estimates of model uncertainty (especially in areas where there is little data to inform the model).

(Remarks on code availability)

Reviewer #2

(Remarks to the Author)
I find that this revised manuscript addresses my initial reservations and concerns and now recommend the manuscript for publication.

(Remarks on code availability)

Open Access This Peer Review File is licensed under a Creative Commons Attribution 4.0 International License, which permits use, sharing, adaptation, distribution and reproduction in any medium or format, as long as you give appropriate credit to the original author(s) and the source, provide a link to the Creative Commons license, and indicate if changes were made.
In cases where reviewers are anonymous, credit should be given to 'Anonymous Referee' and the source.
The images or other third party material in this Peer Review File are included in the article's Creative Commons license, unless indicated otherwise in a credit line to the material. If material is not included in the article's Creative Commons license and your intended use is not permitted by statutory regulation or exceeds the permitted use, you will need to obtain permission directly from the copyright holder.
To view a copy of this license, visit https://creativecommons.org/licenses/by/4.0/

Reponse to reviewer comments for the manuscript entitled "Key link between iron and the size structure of three main mesoplanktonic groups (Crustaceans, Rhizarians, and colonial N_2-fixers) in the upper ocean" submitted to Nature Communications (manuscript NCOMMS-25-50071)

We thank the two reviewers for their insightful comments and suggestions. We have addressed their concerns in the tracked change manuscript and include in this document a point by point response to their comments in blue. The page number correspond to the tracked change word document.

Reviewer #1 (Remarks to the Author):

General comments
The manuscript by Dugenne et al. presents an interesting analysis of image-based databases of mesoplankton abundance, biomass and distribution against environmental parameters using various statistical approaches to provide machine learning predictive biogeographical maps. The article is generally well written, and the data and analysis support the conclusions.

There are no major issues with the article; three more minor comments relate to the presentation of the data (the figures and sections of the text), an estimation of model error where observations are sparse/non-existent, and consideration of a common depth component to their analysis.

Thank you, we believe we addressed the minor comments carefully in the revised draft, following the changes noted below.

Specifically, the article figures include very small text and are difficult to interpret (e.g., what do panels g and h on Fig. 1 represent; are the cake sections and concentric circles providing the same information?).

We have now increased the font for figures 1, 3, 4, 5, and 6.

As noted in the caption, Fig. 1g&h represent the relative proportion of diverse plankton functional groups (color-coded), separated from broad (most inner circle) to fine (outer circles) taxonomic ranks in the scanner (**g**) and UVP (**h**) datasets.

Since image annotations are not always done at the same taoxonomic rank (e.g. phylum, class, family, genus, morphotype), depending on the expertise of the annotator(s) and the image resolution(s), the concentric circles allow to represent the percentage of validated annotations at any given rank, with an expected increased in unassigned labels (represented as empty portions of the circles) towards the finest rank, as it is more difficult to identify images at fine taxonomic ranks.

We have now edited Fig. 1 caption as: …"Diversity of taxonomic annotations (n represent the total number of images in each dataset) separated by taxonomic ranks (red to pink rings) and colored by plankton functional groups in the scanner (g) and UVP (h) datasets. Blank portions correspond to organisms unassigned at the considered ranks, ***increasing in proportion for finer taxonomic ranks.***" L.118-125

The second comment relates to an estimate of model confidence where observations (of

mesoplankton biomass and size-structure) are sparse or even non-existent. It would be good in the main text to see some estimate of the confidence of the model predictions, i.e., Figure 1a and 1 b highlight that there is very little or no data in the Southern Ocean and Pacific Ocean, whereas Figure 2c presents global data. Using the machine learning tools the authors apply, is there a way to add confidence limits to the areas where the model is predicting outside of its parameter space?

Thank you for pointing this, we have addressed this comment by fitting boosted regression models on bootstrapped observations (e.g. randomly sampled with replacement) 50 times and show the uncertainties in model predictions, represented by model coefficient of variations (standard deviation / mean predictions), in the new supplementary figure S2. This is explained on l. 993-994 of the revised Material and Methods section: "50 model predictions were performed on bootstrapped observations in order to estimate model uncertainties represented by the predicted coefficients of variation (Supplementary Fig. 2)."

Patterns are presented in the revised draft on l. 151-153: "Bootstrapped model predictions were generally more variable in regions of peak abundances with fewer PSSdb observations, reflecting larger uncertainties in subpolar and polar ecosystems, for all three mesoplankton groups with highest coefficient of variations ranging between 60% and 120% on average (Supplementary Figure 2)."

The third comment relates to a consideration of the variable depth components included: the biomass and spectral data are from the upper 200 m; many of the iron proxies and physical mixing metrics describe water-column impacts; whereas the productivity proxies (chlorophyll, nutrients, light, phytoplankton community size composition) are for surface waters only. Have the authors considered whether this introduces bias or removes potential linkages between the mesoplankton biomass and the environmental conditions? For example, does surface nitrate concentration reflect the availability of nitrogen for the community across 200 m? Does incidental irradiance on the sea surface reflect the light availability to the community at depth? Could variability in the depth of the productive layer (i.e. euphotic zone) compensate for low surface water metrics? This is not a major negative comment, but a consideration; that strong relationships are found nevertheless highlights that these are only potential considerations.

Thank you for indicating this potential bias in our analysis. We had initially developed the code with the possibility to work with surface or depth-averaged/depth-integrated environmental covariates for model predictions, recognizing that both estimates for covariates are typically highly correlated, but still different. Given that our spectral compilations indeed represent the 0-200m depth layer, we agree the latter could be more relevant in our study, even if colonial N_2-fixers and most classes of Rhizarians show much higher abundance at the surface, and certain Curstacean classes display regular diel vertical migration in the 0-200m layer.

We have updated the manuscript with predictions from the depth-integrated environmental covariates. As expected, the differences in predictions were more significant for the group of Crustaceans and the heterotrophic classes of Rhizarian. Overall, model performances increased significantly for theses groups (from 0.61 to 0.88 for Rhizarians and from 0.69 to 0.79 for Crustaceans), while staying similar for colonial N_2-fixers. We note these differences on l. 907-911: "The scripts allow predictions with both depth-integrated and surface

covariates in case groups were more abundant in the surface layer. In this study, model performances increased significantly with depth-integrated covariates, especially for Rhizarians (from 0.61 to 0.88) and Crustaceans (from 0.69 to 0.79), while staying similar for colonial N_2-fixers as they are typically found in greater abundances in the surface (data not shown)."

Importantly, even if these new models were trained on different, but highly correlated, covariates (depth integrated covariates instead of surface values), we note that the importance of the three iron proxies remain unchanged, as dust, aerosol and total iron are all present in the top-3 features explaining the most variance in mesoplankton spectral biogeography. In particular, dust deposition is now ranked 1st for N_2-fixers observations, surpassing previous ranking. Aerosol also moved up to the 2nd rank for Rhizarians, and total iron remains at the 1st rank, together with chlorophyll concentration, in explaining Crustaceans biogeography.

Specific comments

Ln 16, is fish production an ecosystem service as with C-export and nutrient supply? What do the authors mean by fish production – a socio-economic concept or grazing / trophic transfer potential? (See also Ln 37) Fish production is indeed considered an ecosystem service in this study, both for socio-economic concept and for trophic transfer all the way to apex marine predators.

Ln 17, following a 'however' with a 'how' is confusing. Edited to: "However, the linkages between environmental factors and mesoplankton spectral biogeography remain largely unresolved" (l. 17-18)

Ln 18, not clear at this point of the article as to what 'spectral biogeography' is.
L.19-20 give further explanation: "Using global compilations of Rhizarian, colonial N_2-fixer, and Crustacean images, we reveal the paramount role of iron in shaping the size structure and related biogeography of these groups".
We also now define the term clearly on l. 53-54: "Using this framework, ecologists have shown that most plankton communities varied in size with latitudinal gradients (19,20), following the set of empirical rules and theoretical principles linking global environmental gradients to plankton size structure, here defined as spectral biogeography, reviewed by Barton et al. (21)."

Ln 23, how do the authors know that their explanatory variables are comprehensive? Would it not be better to include some indication of what these variables are as to how they are comprehensive? We've replaced the term "comprehensive" by "extensive" (l.23)

Ln 43, 'certain taxa' and /or certain size classes? Corrected to "certain size classes" (l. 53)

Ln 48, contradictory rather than contradicting. Corrected (l.58)

Ln 49, autotrophic N2-fixers could also include diazotrophic diatoms; better to emphasize that it is only Trichodesmium. Corrected to : "heterotrophic Crustaceans, mixotrophic Rhizarians, and autotrophic N2-fixers ***represented by the genera Trichodesmium***." (l.59-60)

Ln 55-56, individual Trichodesmium cells are only ~10 um so is this paradoxical? We were rather referring to the total length of the filament (l.65-66)

Ln 80, previous line (Ln 66) describes the three groups as keystone, why use main here? Corrected (l.93)

Fig. 3, panel b is confusing with multiple y-axis on the left and right and different colours; please consider a clearer way to present these results. Added the following for clarity:"Main iron proxies, ***aerosol (red), dust (yellow) or total iron (grey)***, linked to the spectral biogeography of colonial N_2-fixers (top panels), Rhizarians (middle panels), and Crustaceans (bottom panels)." (l.393)

Ln 209-212, this sentence is long and confusing, introducing copepods and plasticity in their dietary habits and then mentioning the complications of Trichodesmium taxonomy. Consider splitting as difficult to follow the point being made. We now split the sentence as follow: "Using the same modeling approach applied to finer taxonomic groups, including copepods specific families that often correlate with a certain diet (e.g. omnivores or carnivores) as compiled in Benedetti et al. (41), we highlight the potential adaptations of these subgroups to certain environmental conditions. In the case of Trichodesmium morphotypes, differences in environemental linkages are indicative of plasticity, since multiple species can form either morphotype." (l. 434-437)

Ln 228, How many Phaeodaria and Foraminifera are colonial? The majority according to the Tara dataset included in this paper, and further desicribed in :
Tristan Biard, Lars Stemmann, Marc Picheral, Nicolas Mayot, Pieter Vandromme, et al.. In situ imaging reveals the biomass of giant protists in the global ocean. Nature, 2016, 532 (7600)

We now mention this in the Material and methods on l.1116-1118: "Classes of Rhizaria were almost exclusively found as solitary (Collodaria) or colonial (Phaeodaria, Foraminifera) forms in the epipelagic, so we could not explore possible niche partitioning amongst colonial and solitary Rhizarians"

Figs. 4, 5 and 6; the Venn diagrams (a) in each have many (many) numbers and not clear which are the key values to consider. The text in all panels is tiny (especially the correlation coefficients) and the colours in the too pale to clearly see. We have increased the size and used a bold font for the main numbers to consider in the Venn diagrams, representing the degree of partitioning amongst subgroups for singular environmental factors. The other numbers consider the interactive effects between factors. We increased the font for correlation coefficients, which are also presented in the text for further support. Colors are faded in order to visualize all groups more easily.

Ln 263, does a discussion need a title? We followed the journal format. Since we do not provide a separate conclusion after the discussion, we thought it would be nice to include our main points of conclusion in the header

Ln 277, what is considered the best predictor of ecosystem services? The preceding text (Ln 275 onwards) mentions CMIP models, data compilations and plankton size structure, rather than bulk biomass and productivity estimates – which one is the rest of the (long) sentence referring to? We now split the sentence for clarity: "Indeed, the complexity of plankton functional types represented in the Coupled Model Intercomparison Project 6 models has significantly increased with the support of dataset compilations (e.g MAREDAT, COPEPOD), representing bulk biomass estimates so far. Since plankton functional type and

size structure, rather than bulk biomass and productivity estimates, is considered the best predictor of ecosystem services like fish production or carbon export, our dataset should represent a good opportunity to examine the effects of climate change multi-faceted stressors on essential marine ecosystem services." (l.711-715)

Ln 282, contrasting correlations rather than contrasted correlations. Corrected (l.718)

Ln 302 (and Ln 306), why is upper ocean capitalised (Upper Ocean)? Corrected throughout the manuscript

Reviewer #2 (Remarks to the Author):

I find this to be an extremely interesting and potentially important paper that should, ultimately, be published in Nature Communications. However, I do feel uneasy with the manuscript in its present form in that the vertical resolution of the environmental variables included in the models varies greatly.

Thank you for indicating this potential bias in our analysis. As mentioned in the response to reviewer 1, we have now updated our predictions based on 0-200m integrated covariates when possible.
Model performances increased significantly for Rhizarians and Crustaceans (from 0.61 to 0.88, from 0.69 to 0.79 respectively), while staying similar for colonial N_2-fixers. Even if these new models were trained on different, but highly correlated, covariates (depth integrated covariates instead of surface values), we note that the importance of the three iron proxies remain unchanged, as dust, aerosol and total iron are all present in the top-3 features explaining the most variance in mesoplankton spectral biogeography. In particular, dust deposition is now ranked 1st for N_2-fixers observations, surpassing previous ranking. Aerosol also moved up to the 2nd rank for Rhizarians, and total iron remains at the 1st rank, together with chlorophyll concentration, in explaning Crustaceans biogeography.

The mesozooplankton size spectra and distributions studied cover the upper 200 m of the water column, while a number of the environmental variables included in the models are extracted from satellite products which, typically, are derived from surface optical characteristics and, thus, relate to only a very small proportion of the water column (usually around 10m in temperate regions). Only about 25% of global ocean primary production is associated with the surface 10 m and, in tropical regions, up to ~90% of water column productivity occurs below 10 m.

We had initially developed the code with the possibility to work with surface or depth-averaged or depth-integrated environmental covariates for model predictions, recognizing that both estimates for covariates are typically highly correlated, but still different. In addition to depth integrated chlorophyll concentration, we now have included integrated net primary production products to reflect the disparity in surface versus deeper productive layers. These new covariates are presented on l. 904-909.

The authors express surprise that the satellite products indicating the relative size distribution of the phytoplankton community did not correlate well with their mesozooplankton spectra/distributions but I honestly would not expect the surface community size distributions

to be very important for determining the characteristics of mesozooplankton down to 200 m! Subsurface chlorophyll peaks are dominant features in most of the words oceans and these are often important feeding areas for mesozooplankton. I find the iron correlations intriguing (and a potential game changer in our understanding of plankton ecology. I do not, however, find the lack of convincing correlations with surface phytoplankton community characteristics (and productivity) to support the authors' assertion that iron is more important for determining mesozoopalnkton spectra than the characteristics of the food organisms they depend upon. I believe the authors need to address this discrepancy with respect to vertical scales their environmental variables represent before publication can be recommended.

We agree with this comment and decided to include proxies for deep phytoplankton productivity in our updated models. Predictions show that iron remain the (co)first variable explaining the spectral biogeography of Crustaceans in the upper ocean. Integrated chlorophyll concentration, as rightfully pointed to, now shows an increased importance in resuming these spatio-temporal patterns (updated Table 1).

Further, we note on l.956-960 : "Like Barton et al. (21), we recognize that mesoplankton size biogeography is ultimately determined by the interplay between both bottom-up and top-down (e.g. size-selective predation by fish or other nekton) controls, though the latter are out of this paper's scope as this would require an extensive characterization of larger predators (through space and time) that are poorly detected with the imaging systems we used. Hence our results underscore the importance of specific covariates that may vary with the inclusion of other global gridded datasets."

I am also uneasy with respect to the authors' suggestion that while temperature effects stemming from anthropogenic climate change will likely lead to smaller mesozooplankton, this ecosystem response may potentially be offset by anthropogenic additions of iron to the system. We really cannot from this study derive the relative importance of T and Fe in determining size spectra and, in my experience, conclusions such as those suggested here can be (and often are!) misused by the non-scientific community. I would prefer to see this speculation on the part of the authors removed but that may be a question of personal taste (and too many misrepresentations of my research in the general media).

Thank you for this suggestion. We agree this study is limited in its ability to foresee the changes in mesoplankton size structure in response to all possible environemental pressures. We hope our compilation will be used to inform ecosystem models, as they remain the standard to foresee changes in plankton communitites under different climatic scenario, as stated in the disucssion l. 708-715: "With the predictions of mesoplankton spectral biogeography and associated compilations of pelagic size spectra, expressed as a function of cell abundances or carbon biomass, our hope is to inform the development of biogeochemical and other size-structured models used to foresee the changes in ecosystem functioning and services under future climate change. Indeed, the complexity of plankton functional types represented in the Coupled Model Intercomparison Project 6 models has significantly increased with the support of dataset compilations (e.g MAREDAT, COPEPOD), representing bulk biomass estimates so far. Since plankton functional type and size structure, rather than bulk biomass and productivity estimates, is considered the best predictor of ecosystem services like fish production or carbon export, our dataset should represent a good opportunity to examine the effects of climate change multi-faceted stressors on essential marine ecosystem services."

We also understand that some parts of the abstract could lead to misinterpretation and thus clarify: "Our results suggest that without compensatory mechanisms, future increases in global temperatures could have negative effects on mesoplankton size, possibly limiting carbon export from the productive layers to sequestration depth, that might be offset by expected increases in iron inputs that benefit N_2-fixers, Rhizarians, and eventually Crustaceans." (l. 24-27)

As far as I can determine (hanging on by my fingernails), the statistics used are robust (except as noted that the environmental variables used do not represent the same scales). There are a few typos and grammatical errors, so careful proof-reading will be necessary if/when the manuscript is accepted for publication. As noted above, however, I do not recommend publication unless/until some consideration is given to the value of using surface proxies to represent pelagic processes to a depth of 200 m.

Katherine Richardson, professor, University of Copenhagen

We appreciate the opportunity to further work and improve the manuscript. Following your recommendation, we show that, indeed, integrated covariates resulted in improved model fits and actually increased the relative importance of the different iron proxies in explaining the spectral biogeography of keystone mesoplankton groups. We hope the new manuscript will have carefully addressed both reviewer suggestions and recommendations
